# The Gut-Brain Axis: How Microbiota and Host Inflammasome Influence Brain Physiology and Pathology

**DOI:** 10.3389/fimmu.2020.604179

**Published:** 2020-12-10

**Authors:** Andrina Rutsch, Johan B. Kantsjö, Francesca Ronchi

**Affiliations:** Maurice Müller Laboratories, Department of Biomedical Research, Universitätsklinik für Viszerale Chirurgie und Medizin Inselspital, University of Berne, Berne, Switzerland

**Keywords:** microbiota, gut-brain axis, inflammasome, multiple sclerosis, Alzheimer’s disease, Parkinson’s disease, neuropsychiatric disorders

## Abstract

The human microbiota has a fundamental role in host physiology and pathology. Gut microbial alteration, also known as dysbiosis, is a condition associated not only with gastrointestinal disorders but also with diseases affecting other distal organs. Recently it became evident that the intestinal bacteria can affect the central nervous system (CNS) physiology and inflammation. The nervous system and the gastrointestinal tract are communicating through a bidirectional network of signaling pathways called the gut-brain axis, which consists of multiple connections, including the vagus nerve, the immune system, and bacterial metabolites and products. During dysbiosis, these pathways are dysregulated and associated with altered permeability of the blood-brain barrier (BBB) and neuroinflammation. However, numerous mechanisms behind the impact of the gut microbiota in neuro-development and -pathogenesis remain poorly understood. There are several immune pathways involved in CNS homeostasis and inflammation. Among those, the inflammasome pathway has been linked to neuroinflammatory conditions such as multiple sclerosis, Alzheimer’s and Parkinson’s diseases, but also anxiety and depressive-like disorders. The inflammasome complex assembles upon cell activation due to exposure to microbes, danger signals, or stress and lead to the production of pro-inflammatory cytokines (interleukin-1β and interleukin-18) and to pyroptosis. Evidences suggest that there is a reciprocal influence of microbiota and inflammasome activation in the brain. However, how this influence is precisely working is yet to be discovered. Herein, we discuss the status of the knowledge and the open questions in the field focusing on the function of intestinal microbial metabolites or products on CNS cells during healthy and inflammatory conditions, such as multiple sclerosis, Alzheimer’s and Parkinson’s diseases, and also neuropsychiatric disorders. In particular, we focus on the innate inflammasome pathway as immune mechanism that can be involved in several of these conditions, upon exposure to certain microbes.

## Introduction

The mammalian intestinal microbiota comprises bacteria, viruses, fungi, yeasts, and bacteriophages. This community starts to develop at birth and continues for two–three years, in humans, until it reaches a stable composition (1). However, it continues to be influenced by different environmental and lifestyle factors throughout the lifetime. Therefore, the microbiota composition differs remarkably even between healthy individuals (2). Under healthy conditions, the microbiota influences numerous physiological processes within the host, such as protection against pathogens, nutrient digestion and absorption, development, and education of multiple host organs and the immune system (3–6).

In the last decade, different studies revealed strong associations between changes in the microbiota composition (a situation called “dysbiosis”) and various host diseases (4, 5, 7). Interestingly, among those, there are also diseases affecting host organs in physical distance from the gut (8, 9), like the central nervous system (CNS) (7, 10–22). Moreover, a relevant contribution of gut microbiota is not only restricted to neuroinflammatory and psychiatric disorders (23–32) but also to brain development (13).

The CNS has long been considered an immune-privileged organ. The blood vessels that vascularize the brain are formed by endothelial cells firmly held together with tight junctions building the blood-brain barrier (BBB). The BBB allows to strictly regulate the movements of molecules, ions, and cells between the periphery and the brain (33). Importantly, the BBB protects the brain from pathogens and unwanted immune reactions that could damage the neurons and their connections (34). However, the idea that the CNS is an immune privileged organ has been reconsidered, as the functional immune cells can enter the CNS through the BBB, the choroid plexus, and the lymphatic vessels and have been described beyond neuropathological conditions (26, 35–47). Among the molecules that can pass the BBB are also bacterial products and metabolites shaping not only the CNS development and functions (6, 15) but also the genesis of certain diseases (33–42).

The communication between the CNS, the intestine, and the microbiota happens through the so called Gut-Brain Axis (GBA), a complex bidirectional communication network between the intestine and the CNS (10, 48). This axis involves different pathways such as the autonomic and enteric nervous system, the endocrine system, the hypothalamic-pituitary-adrenal axis (HPA), the immune system, and the microbiota and its metabolites (8, 31, 32). Several neurotransmitters (11, 49) and metabolites such as essential vitamins, secondary bile acids, amino acids, and short-chain fatty acids (SCFAs) (43, 46–51), modulate many immune system pathways (50–56) that in turn influence behavior, memory, learning, locomotion, and neurodegenerative disorders (45, 52–55). Among those pathways, researchers showed that the inflammasome plays a role in depressive- and anxiety-like behaviors, and locomotor activity (57). A potential role of dysbiosis has been suggested as cause of these mood and behavioral defects (55), however, the exact mechanism behind these phenomena still needs to be understood.

Despite growing evidence, a significant gap of knowledge still exists in understanding the exact mechanisms involved in the communication between gut and brain during health and disease. In this review, we provide an overview of the current state of research about the effect of microbiota on the GBA in homeostasis and disease states, with a particular interest in the different bacterial metabolites involved. We further discuss the potential contribution of inflammasomes on the GBA, highlighting the critical open questions that remain in the field.

## Host-Microbial Mutualism in The Gut-Brain Axis: Role of Bacterial Molecules and Metabolites in Development and Health

The microbiota is a community of commensal and symbiotic microorganisms that reach a density of more than 10^12^ cells/g of content in the human large intestine (16). 500 to 1,000 different bacterial species populate the mammalian gut, belonging to the four dominant bacterial phyla Firmicutes, Bacteroidetes, Actinobacteria, and Proteobacteria. A well-balanced beneficial interaction between the host and its microbiota is an essential requirement for intestinal health and the body as a whole. Under healthy conditions, the mucosal microbiota plays a vital role in food digestion, vitamin synthesis, angiogenesis, epithelial cell maturation, development, education of the host immune system, and protection against pathogens (58–66). Notably, the microbiota orchestrates the local immune system in the intestine (67), and shapes immune and non-immune cells located in distal sites and acting systemically (68, 69).

Colonization of the intestine with a unique microbial community starts at birth through the exposure of the infant to the microflora of the vaginal tract and the mother’s skin. The microbiota develops and gets stable by the age of 2 to 3 years in humans (65) and within 3 to 4 weeks of life in mice (70). This early life window corresponds to a period in which several organs of the body go through critical phases of development and growth (71). During this period, the infant’s immune system develops and the host microbiota matures and stabilizes. The microbiota first gets into contact with the immune system on the mucosal sites, shaping the immune tolerance to commensal microbes and establishing mucosal integrity at the same time. Together with these events, also distal organs get affected. The brain, in particular, undergoes dramatic changes within the early life period. Within the first three months of life in humans, its size increases more than 50% from the time of birth, reaching 90% of the size of the adult organ within the first five years of life (72). In this period, neuronal development takes place (73) and it is supported and shaped by maternal microbiota (74–79).

The influence of the intestinal microbiota in neurodevelopment was known since early 2000s. Early experiments using germ-free (GF) or specific pathogen-free (SPF) mice treated with antibiotics, to reduce the microbial diversity within the intestine, showed that several neurological problems occur in mice with reduced or lack of proper mature gut microbiota (7, 10, 14, 15, 34, 80, 81). In details, compared to colonized mice, GF mice showed exaggerated hypothalamic–pituitary–adrenal (HPA) restrain stress reaction (10), impared social behaviors (12, 15, 82), reduced anxiety-like behavior (7, 81–83) and increased motor and rearing activity (80, 84). Consistently, certain altered brain developments and behaviors observed in GF mice could be resolved/improved when new born animals were reconstituted with a diverse and intact flora (73, 82, 83). Antibiotic treatment results in reduced expression of the tight-junction forming proteins, occludin, and claudin-5, in the brain, increased BBB permeability, reduced anxiety-like behaviors, and elevated exploratory behavior and home-cage activity (35). The altered behavioral phenotype was associated with dysregulation of genes and metabolites known to be involved in motor control and anxiety-like behavior pathways, like adrenaline, dopamine, 5-hydroxytryptophan (5-HT), postsynaptic density protein 95 (PSD-95), and synaptophysin (80).

**Figure 1:**
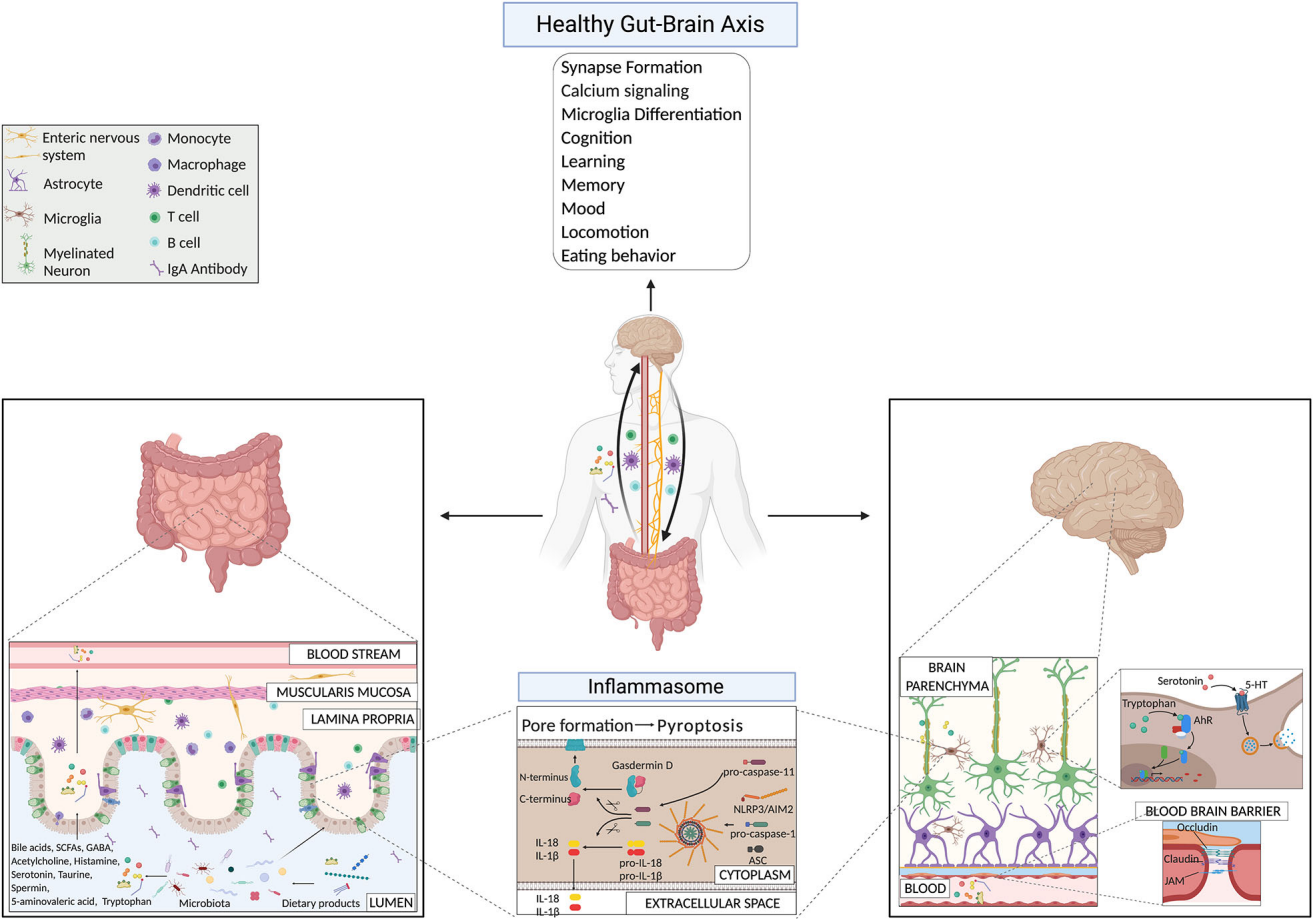
Gut-brain axis mechanisms under physiological conditions highlighting microbial products and the inflammasome pathway.

Lately, it is becoming more evident that microbes can produce neuroactive molecules that directly contribute to the communication between the gut and the brain (**Figure 1**). Neurotransmitters, such as acetylcholine, GABA, and serotonin, produced by bacteria belonging to *Lactobacillus, Bifidobacteria*, *Enterococcus*, and *Streptococcus* species, can influence brain cell physiology directly and indirectly (11, 85, 86). Strikingly, 90% of serotonin required for mood, behavior, sleep, and several other functions within the CNS and gastrointestinal (GI) tract is produced in the gut (87). Binding of serotonin to 5-HT receptors on microglia induces the release of cytokine-carrying exosomes, providing another mechanism for gut-induced modulation of neuroinflammation (88). Another microbial metabolite that influences microglia activity is tryptophan, a serotonin precursor (89). Bacterial metabolites derived from dietary tryptophan could control the CNS inflammation through an aryl hydrocarbon receptor (Ahr)-mediated mechanism acting on microglial activation and the transcriptional program of astrocytes (89). The importance of tryptophan metabolism in maintaining CNS homeostasis was already known a few years earlier, since male GF animals have significantly higher levels of 5-hydroxytryptamine and 5-hydroxyindoleacetic acid in the hippocampus and the serum, compared with conventionally colonized control animals (7). These findings suggest that the systemic circulation could be the route through which the microbiota influences CNS serotonergic neurotransmission. Interestingly, colonizing GF animals post-weaning was sufficient to restore the levels of tryptophan in the periphery and to reduce anxiety in GF animals, but was insufficient to reverse the CNS neurochemical consequences present in adult GF animals (7). This approach highlighted once more the importance of an intact and diverse microbiota from birth on. More recently, it has also been reported that metabolism of tryptophan by activated microglia produces the neurotoxin quinolinic acid, an *N*-methyl-d-aspartate agonist, implicated in several neurological conditions, including Huntington’s disease and depression (90). Recolonizing GF mice with particular bacteria belonging to the Clostridia family, such as *Clostridium tyrobutyricum*, known to colonize the intestinal mucus layer, regulates immune and gut barrier homeostasis through the production of anti-inflammatory metabolites (e.g. butyrate), induces elevation of occludin and claudin-5 levels in brains of GF mice and restores their BBB integrity to the level of SPF mice (91). Furthermore, probiotic supplementations, as *Lactobacillus rhamnosus* (JB-1), in already colonized mice, reduced anxiety- and depression-like behavior in steady-state conditions (92, 93). In 2019, Artis D.’s group showed that SPF mice treated with a cocktail of broad-spectrum antibiotics, GF mice, GF mice recolonized after weaning age with a simple microbiota or a complex microbiota, have defects in fear extinction learning, compared to SPF mice or GF mice colonized with SPF flora at the time of birth (29). Fear extinction learning is a reaction that happens after experiencing an environmental danger and has been implicated in multiple neuropsychiatric disorders, including anxiety disorders like post-traumatic stress disorder (29). The reasons for this altered behavioral response in the absence of a diverse and intact microbiota were reconducted to alterations in pathways involved in synapse formation and calcium signaling at the level of mainly neuronal and microglial cells (29). The researchers showed that the microbiota-mediated changes in synapse formation and fear extinction behavior were not the results of the hypothalamic-pituitary-adrenal axis but of the reduced level of potential neuroactive metabolites (phenyl sulfate, pyrocatechol sulfate, 3-(3-sulfooxyphenyl)propanoic acid, and indoxyl sulfate) in the cerebrospinal fluid, serum and in fecal samples of GF mice compared to SPF mice (29). However, the types of cells (host or bacterial) producing these metabolites are still undiscovered.

From an immunological and metabolomic point of view, GF, SPF mice treated with antibiotics, or gnotobiotic mice with limited microbiome diversity (colonized with ASF for example) showed impaired microglia maturation and immune response upon bacterial stimuli, compared to SPF mice (94). Moreover, the treatment of mice with *E. coli*, isolated from colitic mice, caused colitis and brain memory impairment (95, 96). In contrast, the treatment with *L. johnsonii* restored a healthy gut microbiota composition and attenuated both colitis and *E. coli*-induced memory impairment (95, 96). In addition, bacterial fermentation of indigestible dietary fibers produces among the SCFAs, butyrate, propionate, and acetate in the colon (97). SCFAs maintain gut health by promoting intestinal barrier integrity, mucus production, and supporting a tolerogenic response over inflammation (49, 50, 98, 99). However, their activity is not restricted only to the intestine. A small fraction reaches the systemic circulation and can cross the tightly regulated BBB using their own transporters located on brain vascular epithelial cells (100, 101). SCFAs are, in fact, detectable in low amounts in the human brain under physiological conditions (102). Additionally, they also affect the BBB itself; the colonization of adult GF mice with a complex microbiota or only with SCFAs-producing bacterial strains restores the integrity of the BBB (91). Remarkably, treating GF mice with the oral application of a mixture of the three major SCFAs acetate, propionate, and butyrate, was also sufficient to restore the normal maturation process of the microglia (94). Moreover, SCFAs can modulate neurotransmitters, like glutamate, glutamine, GABA, and neurotrophic factors (103). Propionate and butyrate can influence the cell signaling system *via* modification of the intracellular potassium levels (104), and they regulate the expression levels of tryptophan 5-hydroxylase 1, involved in the synthesis of serotonin, and tyrosine hydroxylase, which is involved in the biosynthesis of dopamine, adrenaline, and noradrenaline (105).

Several other immune pathways have been shown to affect behavior, memory, learning, and locomotion (41, 57, 106–108). Among those, we will discuss the role of the inflammasome pathway in the GBA in more details later on.

## Neurological Diseases: Microbial Effect on The Host Immune and Nervous System

**Figure 2:**
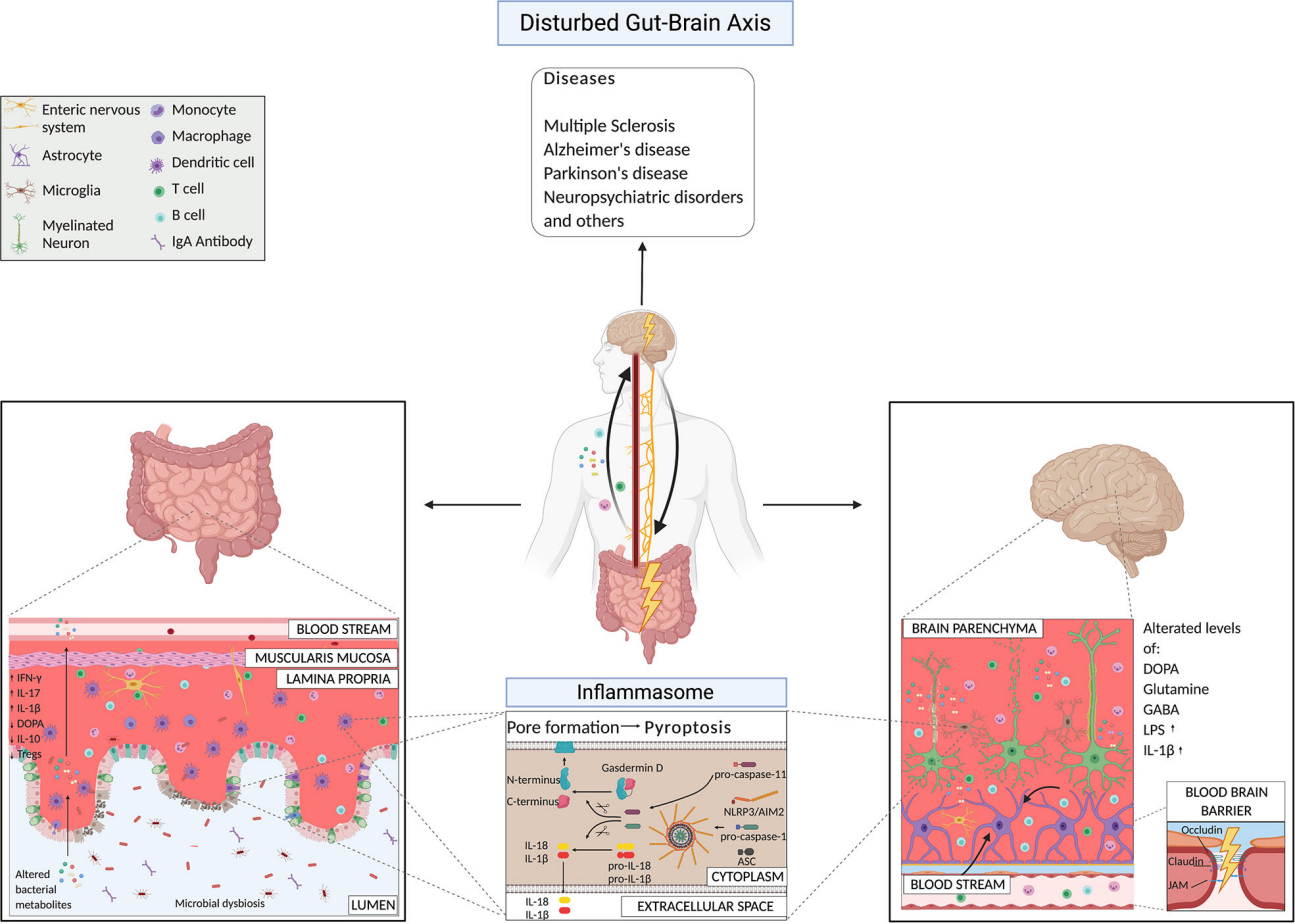
Gut-brain axis mechanisms under pathological conditions highlighting microbial products and the inflammasome pathway.

Several poorly understood environmental factors, including dietary and habit factors, have been linked to susceptibility to neurological disorders and alterations in the gut microbiota (30, 109, 110). The microbiota composition differs significantly between healthy controls and patients affected by neurodegenerative disorders (such as multiple sclerosis (MS), Alzheimer’s (AD) and Parkinson’s (PD) diseases) (20, 22, 23, 32, 111–115), and neuropsychiatric disorders (NPS) (30), like major depressive and mood disorders. Of extreme relevance, the altered microbiota of patients could transfer the disease from a human host to a mouse host (113, 116–120). Here, we present the mechanisms driven by the bacteria that induce different neurological diseases (**Figure 2**). We are at the initial phases of this discovery path, and for the majority of the pathological conditions, we still do not know if the dysbiosis is the cause or rather the consequence of it. Here, we focus our attention on the works that suggested mechanisms of action by bacteria in the etiology of certain CNS disorders.

### Multiple Sclerosis

Multiple sclerosis (MS) is an autoimmune and neurodegenerative disease affecting more than two million people worldwide. This condition is characterized by neuroinflammation, infiltration of lymphocytes into the CNS, demyelination, and axonal loss. Clinical signs associated with MS include ataxia, loss of coordination, hyperreflexia, spasticity, visual and sensory impairment, fatigue and cognitive difficulties. Most of the patients have a relapsing-remitting form of the disease, characterized by a progressive relapse of the symptoms with increased severe neurological deterioration over time (121). The majority of the patients develop lesions in the brain or in both brain and spinal cord, although few develop lesions in the spinal cord only (121). MS is the cause of death in more than 50% of the affected patients (122). Factors involved in the pathogenesis are both environmental and genetic (123–131). Among the environmental factors, microbes (and their secreted products or toxins) play a critical role in the pathogenesis of MS (132, 133). Several relevant pioneer studies showed that external microbial infections and intestinal commensal bacteria can be involved in disease development.

First of all, the microbiota composition of MS patients is different from the one of healthy individuals (104). Interestingly, even MS patients with active disease have an altered microbiota compared to patients in the remission phase, which in turn have a microbiota more similar to healthy controls (25, 134–138). Bacteria belonging to the Clostridia family contribute to the suppression of pathological autoimmunity (134, 139–143). Higher abundance of Firmicutes and the absence of Fusobacteria were associated with a shorter time to relapse in pediatric MS patients (144). Additionally, MS patients that were treated with the antibiotic minocycline, a broad-spectrum tetracycline, have reduced rate of relapses and amelioration of several immunological parameters (such as IL-12p40, metalloproteinase-9, and soluble vascular cell adhesion molecule-1) (145, 146). However, the minocycline effect on the patient microbiota composition was not addressed. In an additional relevant study, three MS patients underwent multiple fecal microbiota transplantations (FMT) to treat severe constipation. This treatment reverted the intestinal illness and improved the MS symptoms (147), proving the existence of a gut-brain connection. Moreover, probiotics supplementations have a therapeutic potential to ameliorate MS, improving the disability status, the mental health, and some inflammatory and metabolic parameters, compared to the placebo group (148, 149). The seminal study by Berer K. and colleagues highlighted the strong immunological *in vivo* impact of the microbiota on MS pathogenesis (112). They recruited a cohort of monozygotic twin couples in which one individual was affected by MS and the other twin was healthy (112). They reported that MS-twins had a higher abundance of taxa like *Akkermansia* in their feces (112). However, the remarkable finding was that transplanting the MS-twins’ intestinal microbes into GF animals, genetically susceptible to developing experimental autoimmune encephalomyelitis (EAE) was enough to promote the disease *in vivo* with a significantly higher incidence than by transplanting the healthy twins’ microbes (112). Interestingly, the immune cells of murine recipients of MS-derived samples produced less IL-10 than cells from mice colonized with the microbiota derived from the healthy twins (112). IL-10 is one of the master regulatory cytokines, and its neutralization in mice colonized with healthy fecal samples increased disease incidence (112). This significant finding highlighted the potential of the human microbiota to induce specific immune system alterations that might be the cause or the effect of the development of MS. However, the exact mechanisms that suppress the production of regulatory cytokines in the hosts receiving certain microbes from MS patients are still unclear. Several investigations in animals started to elucidate these mechanisms. Kasper L.’s group showed that oral but not systemic treatment with a classical pool of broad-spectrum antibiotics (ampicillin, vancomycin, neomycin, and metronidazole) ameliorated the development of acute EAE, in two mouse models (on C57BL/6 and SJL backgrounds) (18). The better disease outcome upon oral antibiotic treatment was associated with a reduction of pro-inflammatory cytokines and an increase in regulatory cytokines like IL-10 (18). This effect suggested that the intestinal microbiota was responsible for the disease severity and the modulation of the adaptive immune response during disease development (18).

One year later, the same laboratory showed how a single bacterium could affect EAE pathogenesis. They used a similar approach in mice by depleting the intestinal bacteria *via* oral antibiotic treatment followed by the reconstitution only with *B. fragilis*, either wild-type (WT) or deficient for the production of the zwitterionic capsular polysaccharide A (ΔPSA) (116). Mice treated with antibiotics alone or recolonized with the PSA-wild-type strain of *B. fragilis* were protected against disease, whereas the mice recolonized with ΔPSA *B. fragilis* and as well the vehicle-treated control group developed the disease (116). The authors also understood that *B. fragilis* induced EAE protection through the generation of IL-10–producing T regulatory (Treg) cells in a PSA-dependent manner (20). They then concluded that oral treatment with PSA could cause protection from EAE in mice, when used as both prophylactic and therapeutic approach, *via* recruitment of CD103^+^ CD11c^high^ antigen-presenting cells and priming of IL-10–producing Treg cells in the cervical lymph-nodes (150). Thereafter, Lee Y. and colleagues showed for the first time that GF mice were almost completely protected from EAE compared to conventionally colonized mice (117). Again, the protection was associated with a decrease of pro-inflammatory cytokine levels, such as IFN-γ and IL-17A, and an increase in Treg cells in the peripheral organs, the gut and the spinal cord (117). Strikingly, GF mice colonized with only a single bacterium, the SFB, developed EAE, and a T helper (Th)-17 pathogenic immune response in the gut and in the CNS (117).

In the recent milestone study from the groups of Gommerman J. and Baranzini SE., IL-10- and IgA-producing plasma cells have been suggested to have a role in ameliorating EAE in mice and to correlate with relapse in MS patients (151). Microbial specific IgA-secreting cells were shown to be present in both the bone marrow and, in particular, in the brain of EAE-affected mice but not in healthy animals. Remarkably, MS patients during active relapse had less IgA specific for intestinal bacteria compared to patients in remission, suggesting the capacity of IgA-producing cells to migrate from the gut to the central nervous system during disease relapses, as in the mouse system (151). The authors also ruled out one of the potential mechanisms mediated by IgA-secreting plasma cells to suppress EAE. Briefly, commensal-reactive IgA-producing cells expressed IL-10, and the production of IL-10 (partially together with iNOS, but not IgA itself) was essential to ameliorate EAE (151). Lately, in the classical acute EAE model, ampicillin administration alone ameliorated the EAE development (152) with a reduction of proliferating CD4^+^ autoreactive T cells. Miyauchi E. and colleagues showed that the *in vivo* treatment with ampicillin induced the complete depletion of *Allobaculum* bacteria from the small bowel. Monocolonization with this bacterium induced the generation of Th17 cells in the small intestinal lamina propria and systemically, and increased severity of EAE, compared to GF mice (152). However, the disease development was less severe than under conventional hygiene conditions. The authors focused then their attention on bacteria homing to the small intestine that could favor the generation and proliferation of autoreactive T cells *via* presentation of cross-reactive antigens. Mice bearing a T cell-receptor (TCR) specific for myelin-oligodendrocyte glycoprotein (MOG) (2D2 mice) showed higher number of CD4^+^ T cells in the small intestinal lamina propria compared to their wild-type counterpart (152). Some candidate mimicry peptides, like UvrABC system protein A (UvrA), were expressed by *L. reuteri*, and aminopeptidase by strains of *Allobaculum* (152). Interestingly, *L. reuteri* monocolonization did not affect the severity of EAE. However, the bi-colonization with *Allobaculum* and *L. reuteri* together activated MOG-specific T cells toward a pathogenic Th17 phenotype and worsened EAE, with demyelination and cell infiltration in the spinal cord (152). This study highlighted the important synergistic potential of several intestinal bacteria, along the entire intestinal tract and perhaps residing even in close contact with the mucus layer, in mediating the disease development through molecular mimicry.

Altogether, these breakthrough murine studies prove that certain intestinal bacterial species profoundly affect EAE pathogenesis, altering the balance between pro- and anti-inflammatory immune responses *via* a direct effect of some bacterial products. Treating mice that spontaneously develop EAE with a cocktail of broad spectrum antibiotics resulted in dramatically different outcomes, depending on whether the antibiotics were given before or after the onset of the autoimmune disease. Prophylactic antibiotic treatment (at 1–3 weeks before the clinical onset of the disease, starting from 2–4 weeks of age) led to a significant reduction of susceptibility to spontaneous EAE, accompanied by altered intestinal microbial composition and a decrease in Th-17 cell development (153). In contrast, antibiotic treatment after the onset of the first signs of spontaneous EAE did not affect the ongoing disease and CNS inflammation (153). The supplementation of a cocktail of five probiotics (IRT5) before or after the EAE onset in rodents is associated with the delay of the disease onset or suppression of the disease progression, respectively (154). The amelioration of the disease upon probiotic treatment was associated with a higher abundance of Treg cells, higher production of IL-10 by CD4^+^ T cells, B cells, and CD11c^+^ cells, together with the suppression of the pro-inflammatory Th-1/Th-17 response (154). These results suggest that microbiota modulation in early life stages or prophylactic treatments in subjects that are genetically prone to develop certain autoimmune diseases, such as MS, could be efficient approaches to ameliorate the disease susceptibility and/or to delay the onset of the disease. Notably, only certain bacteria with specific metabolic or structural characteristics appear effective in preventing or postponing the onset of certain conditions. Such as, oral administration of SCFAs ameliorate EAE development and disease severity (155, 156). Mechanistically, SCFAs favor the enhancement of acetyl-CoA metabolism, histone acetylation, preservation of spinal cord lipid content, suppression of demyelination, oligodendrocyte maturation and differentiation (157, 158). As also mentioned earlier, in the last years, Quintana F.’s group provided an exceptional contribution to the field, revealing the role of dietary tryptophan (Trp) metabolism in MS pathogenesis (118). The gut microbiota metabolizes dietary tryptophan into AhR agonists (119). Firstly, they showed that treating mice with antibiotics or feeding them with a Trp-deficient diet during the recovery phase of the EAE model worsened the EAE scores (118). However, the disease was ameliorated by supplementation of Trp metabolites (like indole, indoxyl-3-sulfate (I3S), indole-3-propionic acid (IPA) and indole-3-aldehyde (IAld)) or feeding with Trp-enriched diet (118). They also found that in patients affected by MS, the circulating levels of AhR agonists are decreased. In conclusion, they suggested a mode of action of Ahr agonists, *via* a direct effect on astrocytes to limit CNS inflammation during EAE (118). A couple of years later, the same group discovered that the microglia has a crucial role in this mechanism of protection from EAE *via* the Trp-AhR pathway (89). Briefly, Trp or I3S treatment ameliorated EAE scores in control mice but not in mice lacking AhR expression on the microglia fed with Trp-deficient diet (89). This treatment was initiated 14 days after EAE induction and ameliorated the disease *via* AhR engagement on astrocytes and microglia (89). Moreover, using primary human microglia, they showed that the *in vitro* stimulation with I3S activated the AhR signaling pathway leading to the suppression of pro-inflammatory pathways (via TNF-α, IL-6, IL-12A, NOS2, VEGF-β expression), and the promotion of anti-inflammatory responses (such as IL-10 and TGF-α expression) (89). Finally, they detected AhR, TGF-α, and VEGF-β expression on myeloid CD14^+^ cells in the demyelinated active and chronic MS lesions of patients (89). These findings suggest that metabolites derived from the digestion of tryptophan by the gut flora activate AhR signaling in astrocytes and microglia and induce immune protection mechanisms in the host that are important to suppress CNS inflammation, in both animal models and perhaps also in human patients. Altogether, these works suggest the efficacy of different interventions aimed to modify the microbiota composition as prophylactic or therapeutic approaches. Therefore, the need of studies that help to understand the mechanism of action of different microbial methods in different patients, bearing a diverse microbiota, becomes a priority for future research in the field.

### Alzheimer’s Disease

Alzheimer’s disease (AD) is the most common cause of progressive dementia that affects nearly 50 million people worldwide. Symptoms affecting memory and thinking become, with time, critically severe compromising even the simplest daily life tasks. Until today, neither a therapeutic nor a prophylactic strategy exists against this devastating neurodegenerative disorder (159). AD is caused by the formation of aggregates of polymerized forms of β-amyloid precursor protein (Aβ) in soluble multimeric and/or insoluble amyloid deposits in the brain, that trigger a cascade of pathological events leading to neurofibrillary tangles, aggregates of hyperphosphorylated tau proteins, formation of neurofibrillary lesions, and ultimately dementia (159). Several microbial factors have been linked to the AD pathogenesis (140, 141), and a few studies suggested alteration in the commensal microbiota and pathogenic infections as potential causes of AD (142).

Stool microbial profile of AD patients display decreased numbers of Firmicutes and Actinobacteria, and increased Bacteroidetes compared to controls. Within the Firmicutes, the families *Ruminococcaceae*, *Turicibacteraceae*, and *Clostridiaceae* were all less abundant in AD patient (160).

APP/PS1 double transgenic mice, which express special neurons in the CNS with a chimeric mouse/human amyloid precursor protein (APP) and a mutant human presenilin 1 (PS1), show a remarkable shift in the gut microbiota composition compared to healthy wild-type mice (161). Fewer Firmicutes (*Akkermansia* and *Rikenellaceaea*) but more Bacteroidetes (S24-7) were identified in conventionally-raised APP/PS1 mice compared to wild-type littermate controls (161). Importantly, GF-APP/PS1 transgenic mice have reduced Aβ levels in the brain and blood and reduced amyloid load compared to conventionally-raised APP/PS1 mice (161). Fecal transplantation from conventionally-raised APP/PS1 mice into GF APP/PS1 hosts dramatically increased cerebral Aβ pathology in the hosts (161). Altogether, these results strongly supported a microbiota involvement in the development of AD in AD-susceptible animal models (161). Additionally, 5xFAD mice (over-expressing in the brain the mutant human APP carrying Swedish, Florida, and London Familial Alzheimer’s Disease (FAD) mutations along with human PS1 harboring two FAD mutations) recapitulate significant AD and fecal microbial composition features that evolve with age (162, 163). At the age of two–three months, when Aβ plaque deposition in the cortex and hippocampus starts, Bacteroides, Firmicutes, and Verrucomicrobia were the most abundant bacterial phyla (163). At the age of seven months, when the signs of synaptic degeneration appear, Firmicutes became the predominant phylum, with a marked decrease in abundance of Bacteroidetes and Verrucomicrobia. These variations over time were absent in wild-type mice, which had a much more stable microbiota throughout the lifespan (163). These changes in microbiota composition were associated with neuroinflammatory markers. In fact, at 2 to 3 months of age, both pro-inflammatory and anti-inflammatory microglia expanded, while in the following months, only the first subset continued to grow, along with the recruitment of Th-1 CD4^+^ proinflammatory cells, reaching a peak at seven-nine months. On the other hand, the anti-inflammatory microglia declined from three to five months and maintained at low levels after that. Importantly, this was the first time that changes in the microbiota composition have been monitored during a neurological disease development suggesting that the bacterial changes could be happening together or before the immunological and neurological changes. The neuroinflammatory modifications were, in fact, strikingly dependent on the microbiota ones, and the depletion of intestinal microbes *via* antibiotic treatment ameliorated the recruitment and priming of anti-inflammatory microglia and Th-1 cells (163). Co-housing or FMT experiments showed that neuroinflammation and cognitive impairments could be transferred from 5xFAD mice to wild-type counterparts (163). The usage of a sodium oligomannate, with known cognition improvement effect in humans, suppressed neuroinflammation, Aβ plaque deposition, and cognition impairment (164). However, from the microbiota point of view, the most striking result of this therapeutic approach comes from the FMT experiments from oligomannate-treated 5xFAD mice into WT hosts that were pre-treated with Aβ aggregate injections to induce AD development (163). The feces isolated from oligomannate-treated 5xFAD mice transferred protection from neuroinflammatory events in recipient animals (163). Metabolomic analyses on the feces of these animals revealed significant changes in amino acid–related metabolism, in particular for the phenylalanine- and isoleucine-related pathways. Phenylalanine and isoleucine can be uptaken by adaptive immune cells, like Th-1 cells (163). In addition, the intestinal microbiota diversity was altered and the levels of SCFAs were reduced in AD mice compared to wild-type control mice. Therefore, modifications of the intestinal flora impact several metabolic pathways in AD mouse models, that could be leading to cognitive defects, amyloid deposition, and intestinal abnormalities (165). Similarly, a recent work showed that ADLP^APT^ mice present community level-alterations in the microbiota compared to wild-type animals (166). The ADLP^APT^ mice carry six human mutations affecting amyloid precursor protein, presenilin-1, and tau protein, and develop an AD-like pathology with amyloid and neurofibrillary tangles (167). Upon fecal microbial transplantations from WT animals into ADLP^APT^ mice, formation of amyloid β plaques and neurofibrillary tangles, glial reactivity and cognitive impairment were ameliorated in the recipients mice (166). Together, these findings highlight the role of gut microbes in the promotion of neuroinflammation in AD progression through alteration in metabolic and immunological pathways.

Probiotics supplementation has been taken into consideration also for AD (168, 169). The human isolate *Bifidobacterium longum* (NK46) was orally administered in 5xFAD mice and induced anti-inflammatory effects (decrease in lipopolysaccharide (LPS) levels, NF-κB activation, and TNF-α expression), changes in the intestinal microbiota composition of the recipients (increase in Bacteroides and reduction in Firmicutes and Proteobacteria phyla), and suppression of Aβ accumulation in the hippocampus (170).

To conclude, an intriguing and relevant role of the oral pathogen *Porphyromonas gingivalis*, the causing agent of chronic periodontitis, has been elucidated in the etiology of AD (120). We think that the mechanisms adopted by this bacterium could be relevant to study as also some commensals could have a similar mode of action. Dominy SS. and colleagues identified *Porphyromonas gingivalis* and the gingipains, the toxic proteases produced by this bacterium, in the brain of AD patients but not in the brain of control patients with no history of any neurological abnormality or condition (120). Gingipains co-localized with neurons and astrocytes and also tau tangles and intraneuronal Aβ in the tissue of AD patients (120). *P. gingivalis* 16S rRNA was detected in both the cerebrospinal fluid and in the cerebral cortex of AD patients (120). The oral *P. gingivalis* infection in mice that are not genetically susceptible to develop AD resulted in the detection of *P. gingivalis* DNA in the brain and remarkably increased production of Aβ deposits (120). On the other hand, mice that received gingipains-deficient *P. gingivalis* or synthetic gingipains inhibitors had significantly less *P. gingivalis* DNA detectable in the brain, less Aβ production, reduced neuroinflammation, and increased number of healthy neurons in the hippocampus (120). For the first time, some bacterial molecules, as gingipains, have been identified as neurotoxic and having a critical role in the generation of an Aβ response *in vivo* (120). These findings also suggest gingipain inhibitors as valuable instrument for treating *P. gingivalis* brain colonization and potentially even the neurodegeneration in AD (120). This breakthrough study also suggested that some bacteria could impact the physiology of the brain or other tissues by reaching them alive, if specific barriers are leaking or certain conditions will happen, or dead, or by releasing soluble factors. More studies are needed to address the exact mechanism through which this is happening and under which circumstances. It could be relevant to study if mechanisms adopted by this bacterium in the oral cavity are similar to the ones used by commensals in other sites.

### Parkinson’s Disease

Parkinson’s disease (PD) is the second most common neurodegenerative disorder, affecting ten million people worldwide (171). It is a progressive nervous system disorder that affects movement. Symptoms start gradually, from a very mild and hardly recognized tremor in one hand, to reach stiffness or slowing of movements, difficulties in walking, accompanied by cognitive and behavior defects (172). PD affects predominantly dopaminergic neurons in the substantia nigra of the brain, leading to a loss in control and coordination of the movements (172). Both genetic and environmental factors have been linked to the etiology of the disease. Genetically, toxic protofibrils formation, consisting of soluble oligomers of α-synuclein, a presynaptic protein, has been involved in the disruption of synaptic functions and in neuronal death. Targeting α-synuclein (α-syn) has been taken into consideration to develop therapeutic strategies in PD and other synucleinopathies (173). However, still nowadays, there is no cure for this neurodegenerative disorder.

As with most of the diseases, PD patients have a different microbiota composition compared to healthy controls or patients affected by other neurological disorders (174–176). Remarkably, PD patients harbored an intestinal flora depleted in SCFAs (mainly butyrate)-producing bacteria, like taxa from Lachnospiraceae family (175, 177–179), and *Faecalibacterium prausnitzii* (175, 180), those with known anti-inflammatory properties. Those butyrate-producing bacteria are also associated with a decrease in the dopamine metabolite and lower quality of life and signs of depression (22). This finding suggests that a PD-typical microbiota composition could be associated with less dopamine production and PD. Another important characteristic of the disease is a disrupted intestinal barrier, which leads to the systemic dissemination of microbial products like LPS and an increase in expression of intestinal pro-inflammatory genes (181). An essential role of the microbiota in PD pathogenesis has been shown in animal models. GF mice colonized with microbiota from PD patients display physical impairments compared to GF mice colonized with microbiota from healthy human donors (111). Importantly, ASO (overexpressing a-synuclein under Thy1 promoter) mouse model of PD develops progressive deficits in motor function as well as in gut motility (182, 183). ASO GF mice show reduced signs of PD and diminished α-synuclein aggregates in the frontal cortex, but not in the cerebellum, compared to their SPF counterparts (103). Antibiotic treatment of SPF ASO mice induced very mild α-syn-dependent motor dysfunction, like in mice born under GF conditions (103). Moreover, colonization of ASO GF animals with SPF flora at five-six weeks of age recapitulated the significant motor dysfunction observed in ASO SPF mice (103). Importantly, the microglia phenotype and also the gastrointestinal function (measured as fecal output) were significantly improved in antibiotic-treated animals but diminished in GF mice recolonized with SPF flora (103). Remarkably, ASO-GF mice treated with SCFAs mixture showed again mature microglia, formation of α-syn-aggregates, GI deficits, and significantly impaired performance in several motor tasks, as ASO-SPF mice (103). Host exposure to dead bacteria was not sufficient to induce the pathogenesis in the ASO-GF mice (103). Moreover, the simple treatment with minocycline was adequate to reduce inflammation, α-syn-aggregates, and improve motor function (103). Altogether, this work suggests that only live bacteria, producing active metabolites, such as SCFAs, could promote inflammation and development of the disease in the ASO model. These findings highlight the fact that the same bacterial metabolite could have a protective effect on specific disease models and a devastating impact on different ones. Therefore, it is vital to understand how these substances work in different conditions. Transferring PD patient-derived feces to GF mice was of high relevance. The differences in the microbiome profile, in SCFAs amount, and motor dysfunctions (increase in propionate and butyrate) were transferred and maintained *in vivo* too (111). In a different PD animal model, the pesticide rotenone-induced mouse model, researchers reported significant changes in the composition of caecum mucosal-associated and luminal microbiota, with a decrease in abundance of *Bifidobacterium* genus. These differences were associated with alterations in the metabolic pathways expressed by the commensal bacteria (184). In the end, in a differently induced model of PD, the repeated oral administration of *Proteus mirabilis* or its derived LPS to 1-methyl-4-phenyl-1,2,3,6-tetrahydropyridine-treated or young mice was sufficient to induce a PD-like phenotype with motor deficit, loss of dopaminergic neurons in the nigra, brain and gut inflammation with disruption of the intestinal epithelial barrier, and formation of α-syn inclusions in the brain and in the colon (185).

### Neuropsychiatric Disorders

Neuropsychiatric disorders consist of cognitive, mental, and behavior disorders, such as schizophrenia, depression, anxiety, stress and bipolar disorders, autism, eating disorders, and epilepsy. In the last decades, the incidence of these conditions increased dramatically, reaching a percentage close to 40% of affected people worldwide. These patients have impaired health and ability to conduct a healthy life, to learn and work, which implies enormous health and economic effort from society. The etiology of these conditions includes genetic predisposition, injuries, infections, and environmental factors, such as the microbiota.

Patients suffering from major depressive disorder (MDD) had increased fecal α-diversity (increased levels of *Enterobacteriaceae* and *Alistipes* but reduced levels of *Faecalibacterium*) compared to drug-responders-MDD patients and healthy controls. The authors, therefore, reported a negative correlation between *Faecalibacterium* and the severity of depressive symptoms (186). An additional study suggested that the administration of probiotics (*Lactobacillus acidophilus, Lactobacillus casei*, and *Bifidobacterium bifidum*) to MDD patients significantly reduced depressive symptoms compared to placebo (187). From a large microbiome study on a Flemish population cohort, some bacteria have been associated with high quality of life, such as butyrate-producing *Faecalibacterium*, *Coprococcus* bacteria, and others with low quality of life and signs of depression, such as Bacteroides enterotype 2 (22). From fecal metagenomic data, the bacterial capacity to synthesize 3,4-dihydroxyphenylacetic acid, a dopamine metabolite, correlates positively with mental quality of life and suggests a potential role of microbes to produce different neuroactive molecules during depression than during healthy conditions (22).

Patients affected by schizophrenia have also an altered and less rich gut microbiota composition with 77 differently expressed operational taxonomy units (OTUs) compared to healthy individuals (23). The fecal samples derived from these patients were transplanted into GF rodents and could transfer schizophrenic-associated behaviors, such as locomotor hyperactivity and decreased anxiety- and depressive-like behaviors, in the recipients (23). The mice that received fecal samples from schizophrenic patients showed several differentially regulated metabolic pathways in the feces, serum, and hippocampus (23). In particular, glutamine and GABA were elevated in the hippocampus (23). Glutamate was decreased in the stool and hippocampus of these mice, compared to mice transplanted with healthy patient’s feces (23).

Regarding human autism spectrum disorder (ASD), studies showing the importance of the microbiota in the pathogenesis are few and mostly inconsistent, with some exceptions concerning the differences observed for bacteria, such as *Prevotella, Firmicutes, Clostridiales*, like *Clostridium perfringens*, and *Bifidobacterium* species (188), between the ASD patients and the controls. Colonizing GF mice with fecal microbiota from patients affected by ASD was sufficient to promote ASD-like behaviors in the animals (113). This approach seemed to be due to a deficit in the production of two bacterial metabolites, 5-aminovaleric acid (5-AV) and taurine, both being weak GABA_A_ agonists, in ASD individuals compared to controls (113). Maternal immune activation (MIA) is a situation in which the maternal immune system gets activated by infections or infections-like stimuli, like LPS, and it features ASD in the offspring. In MIA animal models, scientists highlighted the importance of specific commensal bacteria in ASD protection. Interestingly, offspring coming from MIA dams showed intestinal microbial dysbiosis with 67 different OTUs compared to the control group, dysregulation of the intestinal barrier integrity with increased permeability, as reported already in children affected by ASD (189), and alteration in their metabolomic profile (20). The pure administration of two bacterial strains as probiotic treatment, such as *Bacteroides fragilis* (and *Bacteroides thetaiotaomicron* but not *Enterococcus faecalis*), could improve the gut dysbiosis, intestinal barrier integrity, the metabolic profile of the animals, and the communicative, repetitive, anxiety-like, and sensorimotor behaviors in the MIA model (20). Concerning the metabolites that could be induced by the intestinal bacteria under certain pathological conditions, in the MIA model, 4-ethylphenylsulfate (4EPS), indole pyruvate, serotonin, glycolate, imidazole propionate, and N-acetylserine were enormously increased in the serum of MIA offspring and entirely restored by the single *B. fragilis* treatment (20). Remarkably, the injection of the only 4EPS metabolite in the naïve healthy animals was sufficient to induce anxiety-like behaviors similar to the ones showed by the MIA offspring (20).

## Role of Inflammasomes in The Gut-Brain Axis: Under Physiological Conditions

As mentioned earlier, in mice, the genetic deficiency of caspase-1, the effector molecule of the inflammasome, is associated with a decrease of innate and stress-induced depressive- and anxiety-like behaviors and affects chronic restraint stress response with a possible involvement of the intestinal microbiota (190).

The inflammasome is an innate immune signaling complex that is activated and assembled in response to the presence of pathogens or danger signals. Once activated, it leads to the production of active pro-inflammatory cytokines, such as IL-18 and IL-1β. There are several different inflammasomes. All consist of a “receptor” protein (such as pattern-recognition receptors (PRRs), like NLRs (NOD-like receptors) or TLR (Toll-like receptors), an adaptor molecule called apoptosis-associated speck-like protein (ASC or Pycard), and of the effector molecule pro-caspase-1. Additionally, inflammasome activation can initiate pyroptosis, a fast and pro-inflammatory form of cell death (106, 190, 191). In general, two signals initiate a successful inflammasome activation. The first signal comes from pathogen- or danger-associated molecular patterns (PAMPS/DAMPS) from outside of the cell and induces the transcription of genes encoding for inflammasome components and products. The second signal comes from intracellular danger signals, such as adenosine triphosphate, uric acid, fatty substances that can induce lysosomal damage, or nicotinamide adenine dinucleotide phosphate oxidase- or mitochondria-driven reactive oxygen species production. These processes result in the assembly and activation of inflammasomes (191–193).

In the CNS, inflammasome activation has been mainly linked to neuroinflammatory conditions. As example, it has an essential role in the progression of several neurological disorders like MS, AD, PPD, and NPS (132, 193–197). However, in the work of Wong ML et al., caspase-1–deficient mice were subjected to different behavioral tests, such as forced swim test, elevated plus maze, novelty suppressed feeding test, marble burying test, and open field test (190). Caspase-1–deficient mice, compared to wild-type mice, showed decreased floating time in the forced swim test, decreased anxiety-like behavior as measured by the unaltered open/closed arms time ratio in the elevated plus-maze (190). Latency to feed decreased after 16 hsec of fasting in the novelty suppressed feeding test (190). Caspase-1–deficient mice buried fewer marbles in the marble-burying test (190). In the open field test, the knock-out mice produced less fecal pellets, showed increased locomotion, and performed faster at the rotarod test (190). Upon treating the mice with either the minocycline antibiotic, which suppresses inflammasome activation and alters the microbiota composition, or after chronic restraint stress experiment for 21 consecutive days, the gut microbiota composition of these mice changed, compared with mice which do not undergo these procedures (190). The differences in microbiota composition resemble the ones reported in caspase-1–deficient mice, such as an increase in the relative abundance of *Lachnospiraceae* (198).

However, we would like to emphasize the fact that the influence of the host inflammasome pathways on the intestinal microbiota composition has been extensively discussed by colleagues and us (194, 199, 200). In fact, in different facilities, mice lacking ASC, NLRP6 (199), or NLRP3 (200) fail to show any detectable differences in the composition of their fecal flora compared to wild-type controlled littermates (199), either co-housed or individually housed, contrary to what has been described in other facilities (194). Instead, the maternal-microbiota inheritance or the cage effect had a much more substantial impact on the intestinal bacteria composition than the host inflammasome expression in our conditions (58). In conclusion, additional analyses are needed to understand in which particular hygiene conditions, mice lacking a functional inflammasome show remarkable microbiota alteration that could affect the health of the host. Moreover, IL-1β and IL-18 also have essential roles for physiological functions in the CNS, as they participate in processes of cognition, learning, and memory (201). Especially in the hippocampus and hypothalamus, high abundance of IL-1β has been described in steady-state in rats already in 1990, in regions of the brain conducting essential and conserved brain functions as memory and learning processes (202). In 1998, Schneider H. et al. could show that expression of IL-1β increases during long-term potentiation in neurons, which consists in a synaptic strengthening process implicated in learning and memory (203). However, in a study from 2004, IL-1β administration into the hippocampus induced memory impairments in a conditioning learning task in rats (204). This discrepancy underlies the complex role of inflammasomes and IL-1β in the CNS. Recently, AIM2 inflammasome has been shown to have a role in the normal brain development. It gets activated in presence of DNA damage that occurs at high levels during infections, trauma, but also neurodevelopment upon massive cell death (205). Under physiological conditions, AIM2 inflammasome is activated during neurodevelopment and contributes to CNS physiology acting through gasdermin-D regulation, and not through IL-1β and IL-18 production (205).

We could then speculate that in presence of certain infectious agents, or intestinal bacteria, the dysregulation of these physiological pathways could cause an overactivation of the inflammasome also in the brain and therefore an alteration of the homeostatic mechanisms. The disruption of these immune sensors could then lead to CNS abnormalities and disease conditions. Inflammasome signaling in the CNS has also been reported in the microglia, the brain’s critical innate immune cells (206), in astrocytes (207), perivascular brain-resident macrophages (208), oligodendrocytes (209), endothelial cells (210), as well as in neurons (211). In the intestine, under physiological conditions, constant stimulation of inflammasomes is happening due to the resident trillions of microbes. It has been shown that released IL-18 importantly contributes to maintaining homeostasis in the gut (193). Several microbial factors can activate the intestinal inflammasome, which might have a distal effect on the brain. However, we still need to link all the events together. An evidence for intestinal inflammasome activation and its effects on the brain comes from the discovery that *Salmonella* leucine-rich repeat protein (SlrP) inhibits *Salmonella* virulence and the typical host anorexic response induced by the infection (212). SlrP inhibits the inflammasome activation and IL-1β production in the small intestine, preventing the flux of IL-1β to the hypothalamus *via* the vagus nerve and therefore the influence on the anorexic-feeding program in the CNS (212). It also promotes survival of the host, and from the microbial point of view microbial transmission to other hosts (212). Several microbial metabolites, such as taurine, spermine, and histamine, can affect the NLRP6 inflammasome activation in the gut and play an important role, if properly balanced, in host-microbial mutualism at the level of the intestine (213). Also, bile acids could be sensed by the inflammasome complex and induce their activation. Bile acids are produced in the liver conjugated with taurine or glycine and released in the upper small intestine during the digestion process. Once in the intestine, they can be converted by gut microbiota and affect the host metabolism and immune response. Researchers have identified that an analogue of oxo-12S-hydroxylithocholic acid methyl ester (BAA473), a microbiota-derived bile acid metabolite, the 11-12-oxo-lithocholic acid (BAA485) can induce the production of IL-18, in a pyrin-inflammasome-dependent manner (214). The group of MacKay C. showed that consumption of high-fiber diet, that inevitably has an effect on the host microbiota, or acetate-treatment, induced increased levels of IL-18 (and not IL-1β) in the serum of the animals *via* GPR43 and GPR109A receptors, that are expressed on colonic epithelial cells and activate the K^+^ efflux and hyperpolarization of the cells and the Ca^2+^ mobilization, in an NLRP3- but not NLRP6-dependent manner, under both steady-state and inflammatory conditions (193).

These data show that intestinal inflammasome activation by the microbiota might lead to the production of effector molecules which have an effect in the CNS *via* the vagus nerve (212). This could be one of the innate immune pathway activated by the intestinal microbes with important distal effects also on the CNS, potentially during both healthy and inflammatory conditions.

## Role of Inflammasomes in The Gut-Brain Axis: Under Pathological Conditions

An exact and overall understanding of the functions of inflammasome activation in physiologic and diseased states is of very high importance, as its effects are guiding to both situations and are tightly regulated. Following, we aim to summarize the state of research on the role of inflammasome activation in different CNS pathologies and what is known so far in terms of bacterial influence on the inflammasome pathways during CNS inflammation (**Table 1**).

**Table 1:** Inflammasome-dependent mechanisms in different neurological disorders.

Disorder/Deficiency	Model	Treatment/Method/Genetically modified animals	Findings	Mechanism	References
Multiple sclerosis	MS patients	Analysis of serum samples	ASC and caspase-1 (inflammasome proteins) are potential biomarkers for prediction of disease onset ASC is a potential biomarker for severity of the disease.	ASC and caspase-1 were found to be elevated in the serum of MS patients compared to the control group. IL-1β was found to be decreased. ASC levels were higher in MS patients with moderate disease onset compared to the mild group.	Keane et al. (215)
Multiple sclerosis	MS patients		IL-1β, IL-18, Caspase-1↑ in PBMCs and the CSF.		Inoue and Shinohara (216); Mamik and Power 2017 (217)
EAE	Mouse	MOG-CFA immunization with pertussis toxin (PTX). IL-1RI^-/-^ mice	IL-1 is importantly involved in the pathology of EAE by promoting pathogenic autoantigenspecific ThIL-17 cells, as IL-1RI-/- mice showed a lower incidence of EAE compared to wt mice after immunization.		Sutton et al. (218)
EAE	Mouse	MOG-CFA immunization with pertussis toxin (PTX)	Inflammasome activation in microglia and border-associated macrophages has a main responsibility for the development of EAE. Essential bacterial influence on the release of IL-1β.		Voet et al. (219)
EAE	Mouse	MOG-CFA immunization with enzymatically active and inactive pertussis toxin (PTX)	Inflammasome activation is needed for PTX-induced adhesion of neutrophils on cerebral capillaries and trigger encephalomyelitis in mice.	PTX activates TLR4 signaling in peritoneal myeloid cells, this leads to pro-IL-1β expression and the formation of a pyrin-dependent inflammasome, and active IL-1β. IL-1β stimulated stromal cells to secrete IL-6, which allows leukocyte adhesion on cerebral capillaries. Caspase-1-, ASC- or pyrin-deficient mice developed less severe EAE pathogenesis.	Dumas et al. (220)
EAE	Mouse	MOG-CFA immunization with pertussis toxin (PTX).Pycard^-/-^ mice	Priming of encephalitogenic T helper CD4+ T cell subset was dependent on ASC-dependent IL-1β production.	PTX induced recruitment of monocytes and neutrophils into lymph nodes, where they produce IL-1β. This primes encephalitogenic T helper CD4+ cells which are responsible for the immune reaction and disease development. Priming of these T cells happens in an ASC-dependent production of IL-1β.	Ronchi et al. (113)
Alzheimer's Disease	AD patients		IL-1B, IL-18↑ in neurons, microglia and astrocytes surrounding Aβ plaques.		Griffin et al. (221), Simard et al. (222), Ojala et al. (223), Malaguarnera et al. (224), Öztürk et al. (225)
Alzheimer's Disease	AD patients		NLRP3, ASC, Caspase-1, -5, IL-1β, -18↑ in PBMCs.		Malaguarnera et al. (224), Saresella et al. (226), Bossù et al. (227)
Alzheimer's Disease	Primary mouse cells and cell lines for microglia	*In vitro* stimulation with Aβ amyloids	Fibrillar Aβ amyloids induced IL-1β secretion from microglia via NLRP3 inflammasomes		Halle et al. (228)
Alzheimer's Disease	Primary microglia	*In vitro* stimulation	NLRP3 activity spreads Aβ pathology in a prion-like manner promoting missfolded proteins to aggregate and form plaques.	Extracellular release of ASC particles (=ASC specks) from microglia cells function as danger signals. They bind to Aβ seeds which leads to further aggregation and spreading of Aβ plaques. ASC specks can also be inter- nalized by macrophages where they induce activation of caspase-1 and release of IL-1β.	Venegas et al. (229)
Alzheimer's Disease	Mouse	APP/PS1 mice deficient for Caspase-1, ASC or NLRP3	All these mice had reduced hippocampal and cortical amyloid plaques deposition with ameliorated disease outcome.	ASC-induced seeding leads to Aβ plaques aggregation and spreading. These ASC specks are released after immune activation of microglia.	Venegas et al. (229), Heneka et al. (230)
Alzheimer's Disease	Mouse	APP/PS1 mice. Pharmacological inhibition of NLRP3 inflammasome with MCC950	Aβ accumulation↓, inflammasome and microglia activation↓, neuroinflammation↓, cognitive impairments↓, phagocytotic function of microglia↑.		Dempsey et al. (231)
Alzheimer's Disease			Aβ accumulation activates microglia and promotes production of proinflammatory mediators by them, and also impairs their phagocytic function.		Sarlus and Hedeka (232), Shi and Holtzmann (233)
Alzheimer's Disease	Rat	IL-1β injection into cerebral hemisphere	Aβ-APP proteins overexpression and dystrophic neurite formation in the brain.		Sheng et al. (234)
Alzheimer's Disease	Mouse	APP/PS1 mice with sustained IL-1β↑↑ in the hippocampus	Plaques pathology↓		Shaftel (235)
Alzheimer's Disease	*in vitro* hiNSC- derived cell lines	HSV-1 viral infection	Induces gliosis and inflammation, production of IL-6, IL-1β, IFN-γ → low-grade HSV-1 infection induced an AD-like phenotype		Cairns et al. (236)
Tautopathy	Mouse	Tau 22 mice. Injection of brain homogenates of wt or APP/PS1 mice into the hippocampus of Tau22 mice.	Cleaved Caspase-1, ASC↑ in the brain at the age of 22 month compared to 3 months old Tau22 wt mice.		Ising et al. (197)
Tautopathy	Mouse	Tau 22 mice deficient for ASC or NLRP3. Injection of brain homogenates of wt or APP/PS1 mice into the hippocampus of Tau22 mice.	Tau hyperphosphorylation in CA1 region in Tau 22 wt mice but not in their NLRP3^-/-^ and ASC^-/-^ counterparts. → NLRP3 activation is upstream of Aβ-tau cascade and pathology, in a IL-1β-dependent manner.		Ising et al. (197)
Tautopathy	Mouse	ASC-, Caspase-1-, IL-1R-, Myd88-deficient mice. Injection of fibrillar Aβ into the striatum.	NALP3 inflammasome and the IL-1β pathway is essential for the microglia activation upon Aβ deposition in the brain.	↓Recruitment and activation of microglia and phagocytes to the Aβ injection site in brain of these mice compared to wt mice.	Halle et al. (228)
Parkinson's Disease	PD patients		IL-1β, Caspase-1↑ in the serum and striatum		Mogi et al. (237)
Parkinson's Disease	PD patients and rodents		Fibrillar form of α-synuclein induced NLRP3- and caspase-1-mediated IL-1β secretion in monocytes and BV2 microglia, or via TLR2-signaling in microglia		Codolo et al. (238)
Parkinson's Disease	PD patients and mice	Patients with a PARK2 mutation, PARK2-/-, PINK1-/- mice	Exacerbation of NLRP3 inflammasome activation in microglia and macrophages in PARK2-/- and PINK1-/- mice. This was confirmed in blood-derived macrophages of patients with PARK2 mutation.		Mouton-Liger et al. (239)
Parkinson's Disease	Human dopaminergic neuroblastoma cells		active caspase-1 cleaves α-synuclein which then newly forms α-synuclein aggregates. Neuronal toxicity↑		Wang et al. (240)
Parkinson's Disease	Rat	Intranigral injection of IL-1β with adenoviruses	Chronic expression of IL-1β in substantia nigra induced activation of astrocytes and microglia and progressive death of dopaminergic neurons → motor impairments.		Ferrari et al. (241)
Parkinson's Disease	Rat	LPS-induced and 6-hydroxy-dopamine-induced PD rats. Injection of caspase-1-inhibitor Ac-YVAD-CMK	Inhibition of caspase-1 → NLRP inflammasome signaling proteins↓ and improvment in the number of dopaminergic neurons.		Mao et al. (242)
Parkinson's Disease	Mouse	α-synuclein A53T transgenic PD mice deficient for caspase-1	IL-1β levels in the midbrain↑. Caspase-1^-/-^ → activation of microglia↓		Fan et al. (243)
Parkinson's Disease	Mouse	MPTP-induced PD mice deficient for NLRP3	Dysregulation of NLRP3 inflammasome contributes to the development of MPTP-induced loss of nigral dopaminergic neurons. Circulating dopamine is a NLRP3 inflammasome inhibitor.	NLRP3-/- mice are resistant to the loss of dopaminergic neurons induced by MPTP. This was associated with caspase-1-, IL-1β- and IL-18↓	Yan et al. (244)
Neuropsychiatric disorders	Depressed, bipolar, and ASD patients		↑expression and activity of NLRP3 inflammasome		Alcocer-Gómez et al. (245) Saresella et al. (226) Baroja-Mazo et al. (246)
Neuropsychiatric disorders	MDD patients		↑ NLRP3, caspase-1, IL-1β mRNA and proteins in PBMC		Alcocer-Gómez et al. (247)
Neuropsychiatric disorders	SSD, ASD, OCD, NSSID patients		↑ NLRP3, caspase-1, ASC, IL-1β, IL-1RN, TNF mRNA in PBMC, ↑plasmitic levels of IL-1↑, IL-18, IL-1Ra, TNFα, and IL-6		Hylén et al. (248)
Neuropsychiatric disorders	MDD patients	Antidepressant drugs	Drugs have inhibitory effect on NLRP3-inflammasome activation, reduction in serum levels of IL-1β and IL-18 and protein levels of NLRP3 and IL-1β		Alcocer-Gómez et al. (247)

### Multiple Sclerosis

Inflammasome components and products have been shown to have a relevant role in MS pathogenesis. Indeed, Caspase-1 and ASC have recently been proposed as candidate biomarkers for MS onset (215). IL-1β and IL-18 seem to contribute to the pathophysiology of MS, as they are upregulated together with caspase-1 in peripheral blood mononuclear cells (PBMCs) and the cerebrospinal fluid (CSF) of MS patients (216, 217).

Evidences in animal models also revealed the importance of the inflammasome signaling in microglia and border-associated macrophages during EAE (219). Essential bacterial influence on the release of IL-1β in the classical animal model of active induced EAE was shown by others and us a few years ago (132, 218, 220). In fact, the importance of the usage of pertussis toxin (PTX) to induce the disease development in an mouse model for MS started to be elucidated. PTX is the major virulence factor of *Bordetella pertussis* and needs to be used in conjugation together with the antigen and the adjuvant during immunization. Dumas A. and colleagues reported the effects of PTX-induced IL-β in the recruitment of inflammatory leukocytes into the brain upon upregulation of adhesion molecules on blood–brain capillaries (220). Briefly, PTX, with its ADP-ribosyltransferase active subunit, induced the activation of TLR4 signaling in peritoneal myeloid cells (macrophages and neutrophils), pro-IL-1β expression, and therefore the formation of a pyrin-dependent inflammasome that releases active IL-1β (220). Subsequently, IL-1β stimulated the stromal cells to secrete IL-6, which is known to induce vascular changes required for leukocyte adhesion. In caspase-1-, ASC-, or pyrin-deficient hosts, PTX did not induce neutrophil adhesion to cerebral capillaries and therefore leads to a less severe EAE phenotype (220). In addition, we showed that expression and production of IL-1β (and not IL-18, IL-1α, IL-6, or IL-23) was transiently and shortly increased post-PTX (and not PBS) injections, in the draining lymph-nodes, during the priming phases of the disease model (132). This happened earlier than the appearance of any clinical symptom (132). The toxin induced the recruitment of inflammatory monocytes and neutrophils in the draining lymph-nodes which were responsible of the production of IL-1β in the tissue (132). IL-1β–producing lymphoid myeloid cells were needed for the priming of multifunctional encephalitogenic T cells, characterized by the production of IL-17A, IFN-γ, GM-CSF, and IL-22 (132). The priming of this pathogenic subset of T helper CD4^+^ T cells was dependent on ASC-dependent IL-1β production, and not on IL-12p35 or IL-12p40 (132). Interestingly we have also shown that the signaling of IL-1β both at the level of T and non-T cells was necessary to generate the immune reactions involved in the disease development (132). We therefore suggested that environmental (bacterial) factors can also affect the priming of autoreactive pathogenic T cells providing new insights into the pathogenic mechanisms of MS and other immune-mediated diseases, including neurological disorders.

In summary, the infectious agent products could be crucial in the activation of the inflammasome pathway in MS pathogenesis. Hence, it would be intriguing to study if similar events could be driven by mucosal commensal microbes in order to better manipulate the microbiota and the immune mediated disease pathogenesis.

### Alzheimer’s Disease

Inflammasome and its products have been implicated in AD pathogenesis since a higher expression of IL-1β and IL-18 has been reported in the microglia, astrocytes, and neurons that surround Aβ plaques or in the plasma of AD patients (221–225). The higher expression of NLRP3, ASC, caspase-1, caspase-5, IL-1β, and IL-18 was additionally found in the PBMCs of AD patients (224, 226, 227). In general, patients affected by tauopathies showed elevated levels of cleaved caspase-1 and ASC and mature IL-1β in the cortex (197). NLRP3 inflammasome-mediated neuroinflammation has been importantly implicated in pathogenesis and progression of AD. Fibrillar Aβ amyloids can induce the secretion of IL-1β from microglia, *via* the NLRP3 inflammasome (228, 249). In turn, NLRP3 inflammasome activity leads to the extracellular release of ASC particles that may function as danger signals (246). They were shown to physically bind to Aβ, seed, and then spread Aβ pathology in a prion-like manner by promoting misfolded proteins to aggregate and form plaques (229).

Animal studies confirmed this significant involvement of NLRP activation, as APP/PS1 mice, lacking the expression of ASC, caspase-1, or NLRP3 had significantly reduced hippocampal and cortical amyloid plaque deposition and ameliorated disease outcome (229, 230). Also, Tau22 mice, another model of AD and other tauopathies, had increased levels of cleaved caspase-1 and ASC in their brain at the age of 11 months compared to their 3-month-old counterparts. Consistent with this, Tau22 mice lacking ASC or NLRP3 expression had lower levels of aggregated and hyperphosphorylated tau in the hippocampus, and their typical spatial memory deficits were rescued (197). Injecting brain homogenates from APP/PS1 or wild-type mice into the hippocampus of Tau22 mice, induced tau hyperphosphorylation in the hippocampus of Tau22 wild-type but not Tau22/Pycard^−/−^ or Tau22/Nlrp3^−/−^ deficient mice, suggesting that NLRP3 activation is upstream the Aβ-tau cascade and tau pathology (197). Moreover, NLRP3 activation induces tau hyperphosphorylation and aggregation in an IL-1β–dependent manner. Importantly, researchers also found that ASC-, caspase-1-, IL-1 receptor-, and MyD88-deficient mice had less recruitment of microglia and mononuclear phagocytes to Aβ in the brain as compared to the wild type control after the injection of Aβ into the striatum (228).

In addition, the pharmacological inhibition of the NLRP3 inflammasome reduced Aβ deposition, neuroinflammation, and cognitive impairment in the APP/PS1 AD mouse model (231). Aβ accumulation activates the microglia and promotes pro-inflammatory mediators’ production and impairment of their phagocytic function (232, 233). IL-1β injection into the cerebral hemisphere increases Aβ-APP proteins in wild-type rats (234). All these evidences suggest that the inflammasome activation in AD pathogenesis could be downstream the Aβ deposits formation, and it could then amplify the neuroinflammation linked to the disease. However, IL-1β seems to have an intricate role, since a sustained overexpression in the hippocampus in APP/PS1 mice was shown to reduce plaque pathology (235). A very recent study highlighted the casuality of *Herpes simplex virus type 1* (HSV-1) in the AD development (236). The authors showed that infecting human-induced neural stem cell (hiNSC) lines with HSV-1 *in vitro* induced gliosis and inflammation, including the production of pro-inflammatory cytokines such as IL-1β, IL-6, and IFN-γ. This work, furthermore, showed that low-grade HSV-1 infection induced an AD-like phenotype in brain organoids derived from hiNSCs (236).

### Parkinson’s Disease

Increased IL-1β and caspase-1 have been measured in the serum and the striatum of PD patients (237). The fibrillar, but not monomeric, form of α-synuclein induced NLRP3- and caspase-1–mediated IL-1β secretion in human monocytes and BV2 microglial cell line (238), or *via* TLR-2 signaling pathway in rodents microglial cells (250). Additionally, early-onset PD patients with mutations in two genes encoding for parkins (PARK2 and PARK2), or mice lacking a mitochondrial serine/threonine protein kinase (PINK1) showed an exacerbated NLRP3 inflammasome response in their microglia and macrophages (239).

In animal models, active caspase-1 was shown to directly cleave α-synuclein, which further promoted the aggregation and neuronal toxicity for neurons of this newly-aggregated α-synuclein (240). Moreover, the chronic expression of IL-1β in substantia nigra of rats induced progressive death of dopaminergic neurons and resulted in motor impairments (241). Injection of the caspase-1 inhibitor Ac-YVAD-CMK was shown to reduce the expression of NLRP inflammasome signaling proteins and improve the number of dopaminergic neurons in LPS- and 6-hydroxydopamine-induced PD in rats (242). In the α-synuclein A53T transgenic mouse model of PD (which overexpress the mutant human A53/α-synuclein), elevated levels of IL-1β in midbrain were measured, but when the mice lack the endogenous expression of caspase-1, this significantly reduced the activation of microglia (243). Moreover, NLRP3^−/−^ mice were resistant to the loss of nigral dopaminergic neurons induced by treatment with the neurotoxin 1-methyl-4-phenyl-1,2,3,6-tetrahydropyridine (MPTP), and this was associated with a reduction in caspase-1 activation and IL-1β and IL-18 secretion (244). However, also for PD, it is very unclear if any microbial insult could be the trigger of the deposit formation that induce the inflammasome activation or vice versa (251, 252).

### Neuropsychiatric Disorders

Increased expression and activity of the NLRP3 inflammasome machinery in circulating immune cells of patients affected by depression (245), bipolar disorder (246), and ASD (226), have been reported. However, works aimed to study how the host system changes in these types of psychiatric disorders are still lacking. A study involving a relatively small cohort of MDD patients and healthy volunteers reported that NLRP3 mRNA and protein levels are increased in peripheral blood mononuclear cells in MDD patients, as caspase-1 and IL-1β, and normalized to healthy levels upon antidepressant treatment (245). In a recent work, involving 40 psychiatric patients affected by schizophrenia spectrum disorders (SSD) (including psychotic disorder, schizophrenia, and schizoaffective disorder), ASD, obsessive-compulsive disorder (OCD), or non-suicidal self-injury disorder (NSSID), expression of genes encoding for NLRP3, caspase-1, ASC, IL-1β, IL-1RN, and TNF are significantly increased in peripheral whole blood of psychiatric patients compared to matched healthy controls (248). Also, at the protein level, the amount of plasma IL-1β, IL-18, IL-1Ra, TNF-α, and IL-6 were more elevated in the psychiatric patients compared to the healthy controls (248). In details, patients with SSD had higher levels in IL-18, IL-1Ra, TNF, and IL-6; whereas OCD patients had higher levels of IL-18, IL-1Ra, and TNF, compared to the healthy controls (248). Importantly, these effects were not caused by the presence of functional mutations of inflammasome components or products, which could lead to increased inflammasome activity and cytokine release, neither by the body mass index (BMI), age at disease onset, depression, or treatment with psychotropic drugs (248). Antidepressant drugs can have an inhibitory effect on inflammasome activation, especially on NLRP3 inflammasome, as they reduce serum levels of IL-1β and IL-18 and protein levels of NLRP3 and IL-1β (247). Host metabolites have an inhibitory effect on NLRP3 inflammasome followed by anti-inflammatory cascade and beneficial effect on the brain, with antidepressant action as example. Specifically, the β-hydroxybutyrate (BHB), a physiological ketone body produced by the liver in condition of fasting, low blood sugar, or carbohydrate-free (like ketogenic) diet consumption had an inhibitory effect on NLRP3-inflammasome (253). In rats, repeated subcutaneous injections of BHB attenuated stress-induced IL-1β and TNF-α expression in the hippocampus. The release of IL-1β and TNF-α caused by stress is tightly regulated by NLRP3 inflammasome (254). These findings suggest that BHB exerts antidepressant-like effects, possibly by anti-inflammatory mechanisms that inhibits or are led by NLRP3-induced neuro-inflammation in the hippocampus (255). This study could also suggest a possible future therapeutic usage of metabolites like BHB to treat neuropsychiatric disorders such as stress-related mood disorders.

## Summary and Open Questions

Emerging data about the influence of intestinal inflammation on the nervous system is extremely critical to connect the missing dots between the two organs for better understanding the synergistic communication within the GBA. This review aimed to present how certain bacterial species could shape the host GBA during healthy and disease conditions. Then, it centralized the attention on the known mechanisms of action of bacteria through the production of molecules that can influence the host’s immune and nervous functions. However, in many cases, in both patients and disease animal models, the exact mechanisms of action and signaling pathways activated by the bacteria and their products or metabolites are yet to be discovered. We also presented how the innate immune inflammasome pathway could act, in some cases, as communication tool between the microbes and the CNS, however still many questions are unanswered. Future studies aiming to dissect the exact mechanisms of microbial action are of critical urgency in the field. Translational approaches and more significant clinical trials are utterly needed to understand the temporal and causal relationships between gut microbiota and specific disease development, to evaluate the suitability of the microbiome as a biomarker of disease, and the efficacy of microbial interventions, such as probiotics and FMT protocols, in the patients. Clinical studies, coupled with animal experiments, are needed to precisely dissect the molecular pathways behind the pathogenesis of several disease conditions. This approach would be necessary for examining the mechanisms behind a specific immunological or neurological effect observed in the presence or absence of particular bacterial species in certain pathogenic conditions. In this case, this review highlights the necessity of studies aimed to discover if and how individual bacterial molecules (products or metabolites) are involved in disease progression or protection. We believe that future research in the field must aim to reveal the precise mechanisms behind the pro- and anti-inflammatory responses induced by the microbes. Of crucial relevance will also be to understand what specific bacterial molecules and metabolites exactly do at the CNS level and how they reach the CNS. It is vital to study on which cells they act, which signaling pathways they activate or suppress, in which organ these mechanisms are affected, and whether they affect both the enteric and central nervous systems. Additionally, it will be essential to address how alterations of the host immune or nervous system will affect also the functions of the microbiota, *via* inflammatory mediators and defensive molecules.

The inflammasome is a signaling pathway that might be activated in the presence of certain bacteria and bacterial molecules. As shown, it is involved in several neurological and intestinal homeostatic and inflammatory conditions. Inflammasome products are targets of several therapies used to treat some of the disorders that we have presented here (256). In some cases, it is involved in the pathogenesis of neurodegenerative diseases, such as EAE, upon bacterial exposure (132). However, it is still obscure if and how intestinal microbial alterations, which are associated with every neurological disease, are upstream or downstream of the immunological (like inflammasomes) and neurological dysfunctions. It is necessary to dissect in which microbial conditions specific mechanisms are activated and how. This approach will allow to design more efficient therapies aimed to modulate the microbiota or the host immune responses to ameliorate or cure specific neurological pathologies.

Overall, we think that the exciting and important discoveries here summarized suggest that bacteria, both pathogens and commensals, have the capacity to stimulate the host intestinal tissue and signal to the brain to promote several aspects of the behaviors of the host and the neurological disease pathogenesis. It is now historically the stage in which all the tools and instruments to identify single bacteria and their products are available. It is possible to then follow them in the various host tissues to understand where they go, which cells they can affect, and which pathways they can activate. This mechanistic approach is, at the moment, utterly needed to better understand how the nervous system is influenced by the intestine. We believe that this knowledge will also lead to the understanding of how to develop better interventions and more efficient and personalized therapeutic strategies for patients affected not only by the neurological disorders treated in this review.

## Author Contributions

AR and FR wrote the manuscript and generated figures and tables. JK generated figures. All authors contributed to the article and approved the submitted version.

## Funding

FR is supported by the following funding systems: Helmut Horten Fundation Grant on project “The role of intestinal microbiota in the generation of encephalitogenic T cells”; Biostime Institute Nutrition & Care (BINC)-Geneva grant on the project “The role of microbiota in brain homeostasis during adulthood and early life”; Novartis Foundation for medical-biological Research Grant no. 19C163 on project “Impact of bacterial-derived metabolites on gut-microbiota-brain axis”.

## Conflict of Interest

The authors declare that the research was conducted in the absence of any commercial or financial relationships that could be construed as a potential conflict of interest.

## Abbreviations

CNS, Central nervous system; BBB, Blood-brain barrier; GBA, Gut-Brain Axis; HPA, Hypothalamic-pituitary-adrenal axis; SCFAs, Short-chain fatty acids; SPF, Specific pathogen-free; GF, Germ-free; 5-HT, 5-hydroxytryptophan; PSD-95, Postsynaptic density protein 95; GI, Gastro-intestinal; Ahr, Aryl hydrocarbon receptor; SFB, *Segmented Filamentous Bacterium*; ASF, Altered Schaedler Flora; MS, Multiple sclerosis; AD, Alzheimer’s disease; PD, Parkinson’s disease; NPS, Neuropsychiatric disorders; FMT, Fecal microbiota transplantations; EAE, Experimental autoimmune encephalomyelitis; PSA, Polysaccharide A; Treg, T regulatory; Th, T helper; Trp, Tryptophan; I3S, Indoxyl-3-sulfate; IPA, Indole-3-propionic acid; IAld, Indole-3- aldehyde; Aβ, Beta-amyloid protein; APP, Amyloid precursor protein; FAD, Familial Alzheimer’s Disease; ASO, Overexpressing α-synuclein under Thy1 promoter; α-syn, α-synuclein; MDD, Major depressive disorder; ASD, Autism spectrum disorder; OTUs, Operational taxonomy units; 5-AV, 5-aminovaleric acid; MIA, Maternal immune activation; 4EPS, 4-ethylphenylsulfate; PRRs, Pattern-recognition receptors; NLRs, NOD-like receptors; TLR, Toll-like receptors; ASC, Apoptosis-associated speck-like protein; PAMPS/DAMPS, Pathogen- or danger-associated molecular patterns; SlrP, *Salmonella* leucine-rich repeat protein; PBMCs, Peripheral blood mononuclear cells; CSF, Cerebrospinal fluid; PTX, Pertussis toxin; HSV-1, *Herpes simplex virus type 1*; hiNSC, Human-induced neural stem cell; PINK1, Mitochondrial serine/threonine protein kinase; MPTP, 1-methyl-4-phenyl-1,2,3,6-tetrahydropyridine; SSD, Schizophrenia spectrum disorders; OCD, Obsessive-compulsive disorder; NSSID, Non-suicidal self-injury disorder; BMI, Body mass index; BHB, Beta-hydroxybutyrate; TCR, T cell-receptor; MOG, Myelin-oligodendrocyte glycoprotein.
